# The Role of Pharmacogenomics in Opioid Prescribing

**DOI:** 10.1007/s11864-022-01010-x

**Published:** 2022-08-24

**Authors:** Aaron K. Wong, Andrew A. Somogyi, Justin Rubio, Jennifer Philip

**Affiliations:** 1 Parkville Integrated Palliative Care Service, The Royal Melbourne Hospital, 300 Grattan St, Parkville, Victoria 3050 Australia; 2 Department of Medicine, University of Melbourne, Eastern Hill Campus, Victoria Parade, Fitzroy, Victoria 3065 Australia; 3 Peter MacCallum Cancer Centre, Melbourne, Victoria 3000 Australia; 4 Discipline of Pharmacology, Faculty of Health and Medical Sciences, University of Adelaide, Adelaide, 5005 Australia; 5 Florey Institute of Neuroscience & Mental Health, 30 Royal Parade, Parkville, Victoria 3052 Australia; 6 Palliative Care Service, St Vincent’s Hospital, Victoria Parade, Fitzroy, Victoria 3065 Australia

**Keywords:** Opioid, Analgesics, Cancer, Palliative care, Pharmacogenomic variants, Precision medicine

## Abstract

Pharmacogenomics is increasingly important to guide objective, safe, and effective individualised prescribing. Personalised prescribing has revolutionised treatments in the past decade, allowing clinicians to maximise drug efficacy and minimise adverse effects based on a person’s genetic profile. Opioids, the gold standard for cancer pain relief, are among the commonest medications prescribed in palliative care practice. This narrative review examines the literature surrounding opioid pharmacogenomics and its applicability to the palliative care cancer population. There is currently limited intersection between the fields of palliative care and pharmacogenomics, but growing evidence presents a need to build linkages between the two disciplines. Pharmacogenomic evidence guiding opioid prescribing is currently available for codeine and tramadol, which relates to *CYP2D6* gene variants. However, these medications are prescribed less commonly for pain in palliative care. Research is accelerating with other opioids, where oxycodone (*CYP2D6*) and methadone (*CYP2B6*, *ABCB1*) already have moderate evidence of an association in terms of drug metabolism and downstream analgesic response and side effects. *OPRM1* and *COMT* are receiving increasing attention and have implications for all opioids, with changes in opioid dosage requirements observed but they have not yet been studied widely enough to be considered clinically actionable. Current evidence indicates that incorporation of pharmacogenomic testing into opioid prescribing practice should focus on the CYP2D6 gene and its actionable variants. Although opioid pharmacogenomic tests are not widely used in clinical practice, the progressively reducing costs and rapid turnover means greater accessibility and affordability to patients, and thus, clinicians will be increasingly asked to provide guidance in this area. The upsurge in pharmacogenomic research will likely discover more actionable gene variants to expand international guidelines to impact opioid prescribing. This rapidly expanding area requires consideration and monitoring by clinicians in order for key findings with clinical implications to be accessible, meaningfully interpretable and communicated.

## Introduction

### What is pharmacogenomics and how does it apply to opioid prescribing

Pharmacogenomics is an increasingly important and effective method used to guide objective, safe, and effective personalised prescribing [1, 2]. Although often used interchangeably, “pharmacogenomics” refers to the impact of multigene variations in DNA and RNA on drug response, whereas “pharmacogenetics” relates only to DNA-based genetic variation. It is now well understood that standard effective medication doses for certain patients will be ineffective for some and cause harm in others [1]. Pharmacogenomic information circumvents this issue by examining how naturally occurring genetic variants and/or gene expression profiles affect response to medication, thus allowing clinicians to select specific medications to achieve maximal efficacy with minimal harm based on a person’s genomic profile [3, 4].

This has revolutionised oncology treatments, where being able to test for tumour mutations (e.g. epidermal growth factor receptor mutation) and patient variants (e.g. dihydropyrimidine dehydrogenase) have allowed clinicians to prioritise the most efficacious treatments upfront and avoid potentially effective treatments that may cause significant harm [3]. In treating infections, sequencing the pathogen and host genome can also reveal susceptibility to treatment response [5]. For example, sequencing the pathogen for drug resistance (e.g. human immunodeficiency virus) and testing the host for markers of drug response (e.g. interleukin-28B for treatment response in hepatitis C virus infection) for efficacy and safety are used to target medicines for greatest net benefit [6–8]. In the past, the administration of such treatments was largely trial-and-error, resulting in low drug efficacy or significant toxicities. In palliative care, this empirical trial-and-error approach remains the standard of care.

A core task in palliative care is to manage symptom needs of patients with life-limiting illnesses. Opioids are among the most commonly prescribed analgesics in cancer care [9]. Almost all cancer patients who undergo surgery receive opioids perioperatively. Up to 60% receive opioids at some point in their cancer treatment [10–12], and 80% with advanced cancer report moderate to severe pain [13] for which opioids are recommended. Despite their prescription frequency, one cannot predict which patient will receive optimal net clinical benefit to a particular opioid. Using the current trial-and-error approach, some patients may receive minimal or no benefit, while others experience significant adverse effects such as delirium, nausea, and somnolence [14]. In the advanced cancer population where prognosis is short, there is little time to waste experiencing unnecessary morbidity from side effects, and an experimental approach to prescribing for pain is particularly undesirable. A personalised treatment plan is critical in this setting. A systematic review of cancer pain found that 32% of cancer patients were undertreated for their pain [15]. A simple blood or saliva genetic test that could determine the strong likelihood of clinical benefit or adverse effects from a certain opioid would provide an opportunity to significantly improve patient pain and symptom outcomes.

This narrative review aims to evaluate and demonstrate the potential applicability of pharmacogenomics within the palliative care population, by summarising current knowledge on opioid pharmacogenomics research in relation to known drug-gene pairs and their potential clinical utility. There is currently a lack of clinician awareness regarding evidence-based pharmacogenomic data, and how to apply the results of tests to patient care [16]. We write for a non-expert audience, using terms understandable to the clinician, and explaining technical language to aid the next step in the translation of pharmacogenomics into this field.

### Genes, alleles, and variants

DNA (deoxyribonucleic acid) consists of 4 chemical bases — adenine, cytosine, guanine, and thymine — which pair to form a double-stranded DNA helix [17]. These base pairs number approximately 3 billion and are organised into 22 pairs of autosomes (chromosomes 1–22) and the sex chromosomes (X and Y) [18]. A gene is a unit of hereditary information at a fixed location (locus) on a chromosome [19]. Every individual usually has two copies of each gene, with the exception of genes on X and Y chromosomes, and each copy is referred to as an allele. Individuals can have the same (homozygous) or different (heterozygous) DNA sequence for both alleles. Genes undergo transcription (converting DNA to RNA) and translation (converting RNA to protein, such as a receptor or enzyme). Humans have ~20,000 protein-coding genes [20], which account for around 1% of the human genome sequence.

Gene variants can be inherited (germline) or acquired (somatic) during a person’s lifetime through environmental factors. Although > 99% of the DNA sequence is shared between any two unrelated individuals, this also means there are several million differences.

Gene variants implicated in pharmacogenomics are commonly inherited via single nucleotide polymorphisms (SNPs), which are small single-nucleotide changes in DNA sequence, and the commonest type of genetic variation [21]. While most SNPs have no impact on protein function, some can predispose to disease and/or drug response (phenotype) and can have a preponderance in certain ancestral groups. Candidate gene variants are either associated with, or known to cause disease, or a drug response phenotype of interest [22]. Actionable variants refer to gene variants that cause a clinical phenotype that reliably influences a person’s response to a particular drug [23], informing the prescriber on clinical actions to be taken.

### Open-access pharmacogenomic databases

There are many open-access genomic databases that synthesise complex evidence-based information relating to gene variants and their function at the molecular and cellular level, in health and disease. These repositories provide in-depth information, from the details of a SNP of interest to its worldwide population frequency distribution (which is increasingly important with the recognition of population diversity due to unrepresented populations), and its phenotypic consequences. Given the rapid advances in gene technology, these databases are updated frequently, some as often as daily [24]. Ready comprehension of these databases, however, requires detailed knowledge of basic science, and are largely used by those with expertise in genomics (e.g. pathologists, geneticists, scientists, and variant curators) or drugs (e.g. clinical pharmacologists and clinical pharmacists), rather than by prescribing clinicians.

The US Pharmacogenomics Knowledge Base (PharmGKB) is one such database and contains systematically organised summary information about the impact of gene variants on drug response [19]. This information is curated in real time from published research and pharmacogenomic-based drug dosing guidelines, which means PharmGKB displays substantial information ranging from unvalidated to strong gene-drug associations, and is organised using specific definitions on levels of evidence for each association [25]. Another important database is the Clinical Pharmacogenomics Implementation Consortium (CPIC), which was formed to aid clinical translation of pharmacogenomics. It contains clinically relevant pharmacogenomic variants and develops guidelines for gene-drug pairs. It is recognised as an international authority in determining clinically actionable variants [16] and is used by many diagnostic pathology services. Both databases were created in response to low uptake of pharmacogenomic translation into clinical practice due to a lack of centralised curated quality resource to bring laboratory results into actionable prescribing [25, 26]. Each database assigns its own hierarchy of evidence to the summarised gene-drug literature to clarify the roles of each variant towards a particular drug, and are two commonly cited resources for clinicians on updated evidence. This article will focus on gene variants that are associated with opioid response with recommended prescribing action based on these two databases.

## Methods

**Fig. 1:**
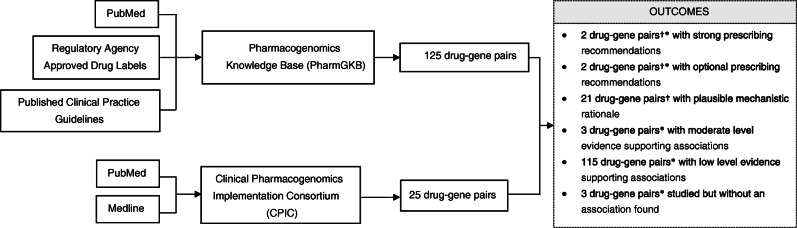
Search method for opioid drug-gene pairs. † assigned by CPIC; * assigned by PharmGKB.

To satisfy the aim of this paper in summarising current knowledge on opioid pharmacogenomics research in relation to prescribing action, we conducted our search on PharmGKB and CPIC (Fig. 1). Both groups have specialised teams that curate information on drug-gene pairs and prescribing recommendations from MEDLINE and PubMed articles, published clinical practice guidelines, and regulatory agency approved drug labels. Using this, we complied a list of candidate gene variants and innate immune markers relevant in responses to opioids.

Search terms included opioids of interest (buprenorphine, codeine, fentanyl, hydrocodone, hydromorphone, methadone, morphine, oxycodone, tapentadol, tramadol, opioid(s)) and phenotype descriptions (analgesia/toxicity, cancer pain, neuropathic pain, nociceptive pain).

Each database was accessed on 01 March 2022. CPIC is updated in real time, whereas PharmGKB were last updated on 05 February 2022. These databases together form large, internationally recognised central annotated databases on the impact of gene variants on pain and opioid drug response.

The RAMESES publication guideline for narrative reviews was used. A narrative review was chosen to comprehensively consolidate updated research in this area, while allowing a more thorough narrative-style discussion on its applicability to palliative care, to build an intersect between both fields. Despite limitations to reproducibility and bias, the available results represent a distillation of widely curated data by established teams of worldwide specialists in pharmacogenomics.

## Results and discussion of the implications

The compiled gene list (Table 1) consists of all opioids of interest (buprenorphine, codeine, fentanyl, hydrocodone, hydromorphone, methadone, morphine, oxycodone, tapentadol, tramadol). It contains 58 genes (some of which have multiple SNPs and/or functional variants) that have been studied in the field of opioid pharmacogenomics from the two curated pharmacogenomics databases CPIC and PharmGKB. Of these, there are 118 drug-gene pairs. There are 9 additional SNPs involved with opioid pharmacogenomics that are not associated with any gene. Of these, there are 11 drug-SNP pairs.

**Table 1:** CPIC recommendations and PharmGKB evidence for opioid drug-gene pairs (as of April 2022)

<Gene/drug>	Codeine	Tramadol	Tapentadol	Buprenorphine	Morphine	Oxycodone	Fentanyl	Hydrocodone	Hydromorphone	Methadone
ABCB1	x/3	X/3			X/3	X/3	C/3		−	X/3
CYP2D6	A/1A	A/1A			−	C/2A	X/3	B/1A	−	C/3
CYP2B6	−	X/3			−	−	−		−	B/2A
OPRM1	C/3	C/3		C/3	C/3	C/3	C/3	C/3	C/X	C/3
COMT	C/X	C/3		C/3	C/3	C/3	C/3	C/X	C/X	C/3
ABCC3					X/3					
ADAMTSL2				X/3						
ADRB2							X/3			
ARRB2		X/3		X/3			X/3			
ALDH5A1										X/3
ANKK1										X/3
ASTN2							X/3			
ATF2							X/3			
BDNF										X/3
CACNA1E							X/3			
CCL11										X/3
CDH2										X/3
CNR1										X/3
CYP2A7P1		X/3								X/3
CYP2C9										X/4
CYP2C19										X/4
CYP3A4				X/3		X/3	X/2A			X/3
CYP3A5						X/3	X/3			X/4
DAO										X/3
DRD1										X/3
DRD2										X/3
FAAH					X/3					
GAD1										X/3
GNB3										X/3
HCN1		X/3								
ICA1		X/3								
IL1B					X/3					
KCNJ6					X/3		X/3			X/3
MYD88							X/3			
NECTIN4										X/3
NGF										X/3
NPIPB8			X/3							
NTRK2										X/3
OPRD1		X/3		X/3		X/3	X/3			X/3
OPRK1					X/3					X/3
OPRL1										X/3
P2RX7					X/3		X/3			
PNOC				X/3						X/3
RFPL4B		X/3								
RGL4		X/3								
RHBDF2					X/3	X/3	X/3			
SEPTIN3	X/3	X/3				X/3		X/3		
SLC22A1		X/3								
SLC6A4				X/3	X/3					X/3
SULT1A1			X/3							
SULT1A3		X/3	X/3		X/3					
SULT1A4					X/3					
TAOK3					X/3					
TLR2					X/3					
TPH2										X/3
TXNRD2										X/3
UGT2B7	X/3				X/3	X/3	X/3			X/3
WBP2NL	X/3	X/3				X/3		X/3		
rs7205113				X/3						
rs62368105				X/3						
rs6973474				X/3						
rs2952768				X/3	X/3		X/3			
rs13169373				X/3						
rs13093031							X/3			
rs6848893						X/3				
rs6961071							X/3			
rs17180299										X/3

*Note: Certain genes have a number of variants that may have different levels of evidence for each opioid. Only the variants with the highest levels of evidence for that particular gene-opioid combination are reflected in this table.*

CPIC evidence: level A or B = prescribing action recommended based on genetic information; level C = no prescribing action recommended; X = not available in CPIC; PharmGKB evidence: level 1A = medical society–endorsed pharmacogenomics guideline available; level 2A = moderate evidence of an association, which has been replicated; level 3 = low level of evidence supporting an association (single or conflicting study, or preliminary evidence); level 4 = studied but no association found at present

There are 2 drug-gene pairs (codeine-CYP2D6 and tramadol-CYP2D6), which consist of clinically actionable variants with strong recommendations to guide/alter prescribing (CPIC level A/PharmGKB level 1A). Another 2 drug-gene pairs (hydrocodone-CYP2D6 and methadone-CYP2B6) have optional prescribing actions (CPIC level B, PharmGKB levels 1A and 2A, respectively). Finally, there are 21 CPIC level C drug-gene pairs (PharmGKB level 3 or unassigned), meaning that there are no current recommended prescribing actions to these; however, there are published studies of varied evidence levels, and plausible mechanistic rationale. The remaining pairs in Table 1 have no CPIC recommendation and represent drug-gene pairs or drug-SNP pairs that either have low level evidence supporting their association or have been studied but without an association found.

### Current evidence on pharmacogenomics and opioids

In examining individual response to opioids, pharmacogenetic effects have been observed for opioid transporters (e.g. P-glycoprotein), receptors (e.g. OPRM1), signal transduction pathways (e.g. ANKK1), drug metabolising enzymes (e.g. CYP2D6), and neurotransmitter enzymes (e.g. COMT). Despite many putative functional gene variants having been identified, most of these still require further validation in larger human samples, and thus, few are considered clinically actionable — meaning that a small number can be used to guide or alter drug therapy [27].

### CYP2D6 (cytochrome P450 family 2 subfamily D member 6)

The strongest evidence with actionable opioid variants relates to the *CYP2D6* (cytochrome P450 family 2 subfamily D member 6) gene. *CYP2D6* codes for the CYP2D6 enzyme that is expressed mainly in the liver and metabolises about 25% of common medications [28]. It is the predominant pathway of metabolism for common important groups of medications in palliative care, including certain antiemetics, serotonin selective reuptake inhibitors, tricyclic antidepressants, and antihistamines [29]. Codeine, tramadol, oxycodone, and hydrocodone account for a large proportion of commonly used opioids worldwide, and each are metabolised to some extent by CYP2D6 [30].

CYP2D6 enzyme function (metaboliser phenotype) is determined by the combination of each person’s inherited alleles. These combinations are assigned into 4 phenotype categories — poor metabolisers (PMs), intermediate metabolisers (IMs), normal (extensive) metabolisers (NMs), and ultrarapid metabolisers (UMs) [31, 32], which determine the wide range of enzyme activity between humans. Globally, NMs are the most prevalent (43–67%), but CYP2D6 allele frequency distributions differ between different subpopulations and ethnicities [28, 33–36]. In 2017, Gaedigk et al. [29] published an important study on genotype-predicted phenotypes by examining CYP2D6 allele-frequency data in different populations. More recently, Koopmans et al. [37] conducted a meta-analysis on > 300 studies to provide estimates of CYP2D6 variation. The global average of people who are CYP2D6 non-normal was estimated at 36.4%, with this being higher in certain countries (e.g. > 50% in Algeria, Argentina, and France were estimated as CYP2D6 non-normal) [37]. PMs were commonest in the British population (12.1%), and least common in East Asians and Oceanians (0.4%) [29]. UMs were commonest in North Africans (40%) [37], and least common in East Asians (1.4%) [29]. These figures demonstrate a vast difference in allele frequency between different groups, highlighting that CYP2D6 gene variants affecting a particular drug can have large differences between populations. CYP2D6 has over 149 alleles whereby some variants may only affect response to one drug or opioid, but not another [30].

The reporting of CYP2D6 phenotype has varied slightly between laboratories and international clinical guidelines. A recent CYP2D6 Phenotype Standardisation Project achieved consensus among CYP2D6 experts in order to standardise genotype-phenotype reporting [26].

The current method of ascribing metaboliser status, and thus, downstream phenotype responses for CYP2D6 alleles, is via a calculated “Activity Score” [30]. Each allele is given a score, where the higher the score, the more rapid the metaboliser status. As each person has 2 inherited alleles to form a diplotype, the score from each allele is then combined additively to form the final activity score, which determines the final metaboliser status.

Phenotypically, being a CYP2D6 UM can have significant implications. In 929 patients, CYP2D6 UMs had increased risk of hospital presentations over a 10-year period compared to other phenotype groups [38]. A consortium of US medical research organisations that assessed 82 pharmacogenes in 5000 people discovered that 96% of people had clinically actionable variants from various genes [39]. These data strengthen the case for using pharmacogenomics in the clinic as most people are likely to have an actionable variant, where the CYP2D6 UM phenotype group predicts poorer health outcomes. Using pharmacogenomic data as a biomarker to guide opioid prescription is likely to not only improve analgesia and reduce side effects but also reduce patient and government costs relating to hospitalisations, and reduce requirements for other drug treatment.

### Codeine and tramadol have actionable variants

Currently, codeine and tramadol are the two opioids with clinically actionable gene variants supported by international guidelines on drug dosing alteration (Table 2). Both medications are prodrugs that need to be metabolised into their pharmacologically opioid active components (morphine and O-desmethyltramadol (M1), respectively).

**Table 2:** Clinical Pharmacogenetics Implementation Consortium (CPIC) guidelines for actionable mutations on codeine and tramadol for CYP2D6

Opioid	CPIC level/PharmGKB evidence**	Phenotype	Phenotype	Guidelines
Codeine	A/1A	Ultrarapid metaboliser	Increased conversion to morphine even at low doses causing increased risk of toxicity	Strong recommendation to avoid use due to potential for toxicity; use alternative non-tramadol opioid. Be alert to adverse effects
		Extensive metaboliser	Normal conversion to morphine, however unpredictable variability leading to issues similar to ultrarapid metabolisers	Strong recommendation to use label-recommended dosing
		Intermediate metaboliser	Reduced conversion to morphine, usually no clinical analgesic significance	Moderate recommendation to use label-recommended dosing. If lacks efficacy, consider alternative non-tramadol opioid
		Poor metaboliser	Reduced conversion to morphine causing reduced analgesia, with persistent central adverse effects (sedation, nausea, xerostomia)	Strong recommendation to avoid use due to lack of efficacy; use alternative non-tramadol opioid. Be alert to insufficient pain relief
Tramadol	A/1A	Ultrarapid metaboliser	Increased conversion to O-desmethyltramadol (M1) leading to increased risk of toxicity (e.g. nausea, vomiting, constipation, respiratory depression, confusion, and urinary retention)	Strong recommendation to avoid use due to potential for toxicity; use alternative non-codeine opioid. Be alert to adverse effects
		Normal metaboliser	Normal conversion to M1	Strong recommendation to use label-recommended dosing
		Intermediate metaboliser	Reduced conversion to M1 leading to reduced analgesia	Optional recommendation to use label-recommended dosing. If lacks efficacy, use alternative non-codeine opioid. Be alert to lack of efficacy
		Poor metaboliser	Greatly reduced conversion to M1 leading to reduced analgesia	Strong recommendation to avoid use due to lack of efficacy; use alternative non-codeine opioid. Be alert to insufficient pain relief

**CPIC evidence: level A or B = prescribing action recommended based on genetic information; level C = no prescribing action recommended; PharmGKB evidence: level 1A = medical society–endorsed pharmacogenomics guideline available; level 2A = moderate evidence of an association, which has been replicated; level 3 = low level of evidence supporting an association (single or conflicting study, or preliminary evidence); level 4 = studied but no association found at present

Codeine is primarily used for its constipating and anti-tussive activity in palliative care and has relatively weak analgesic effects, binding weakly to the mu opioid receptor [40]. Approximately 80% of codeine is metabolised into inactive excreted metabolites [41]. Up to 15% of codeine is metabolised to morphine, its most active metabolite, by CYP2D6. This is the primary method of achieving analgesia through codeine [42], as morphine has a 200-fold higher affinity for the mu opioid receptor than codeine. Morphine is then glucuronidated into morphine-6-glucuronide and morphine-3-glucuronide, where the former provides analgesia and the latter is thought to cause neuroexcitatory adverse effects [43].

Codeine PMs (e.g. CYP2D6 *4/*4 diplotype) have peak plasma morphine concentrations that are 95% lower compared to NMs or IMs [30]. This translates to reduced analgesia and reduced side effects of constipation, as both are mediated via mu opioid receptor activity [44].

In contrast, UMs (e.g. CYP2D6 *1/*2xN diplotype) have increased metabolism/clearance of codeine to morphine. This translates to an increased response to morphine, with approximately 50% higher plasma morphine and metabolite concentrations compared to NMs [30]. This results not only in a more significant analgesic response but also greater adverse effects due to the conversion into toxic morphine glucuronide metabolites, responsible for its central adverse effect profile (sedation, respiratory depression, dizziness, nausea, agitation) and constipation [43]. Specific examples include an increased risk of significant respiratory depression or death in the paediatric population who use codeine post adenotosillectomy [45] and central nervous system depression in infants exposed to codeine through breast milk [46].

For these reasons, guidelines strongly recommend avoiding codeine use in CYP2D6 PMs (due to reduced analgesic response) and CYP2D6 UMs (due to risk of serious toxicity). Although these guidelines are built around use in pain management, a similar practice would make sense when using codeine to reduce cough or frequency of bowel actions.

Tramadol is metabolised by CYP2D6 into its major metabolite responsible for analgesic effects, O-desmethyltramadol (M1), which has 200-times greater affinity for the mu opioid receptor than tramadol [47, 48]. PMs have approximately 40% lower concentrations of M1 and have been shown in prospective clinical trials to experience poor analgesia [48–50]. UMs not only have greater analgesia but also greater toxicity. CPIC guidelines suggest that ultrarapid and poor metabolisers should be prescribed an alternative non-codeine opioid [30].

Several case studies also report that PMs require higher oxycodone doses, whereas UMs experience greater analgesic effect and toxicity due to increased metabolism to oxymorphone [51–56]. Currently, due to the weak and limited evidence, the CPIC consortium recently concluded that data are not yet adequate to allow calculations for dose adjustment. The accepted convention is that UMs have increased metabolism to oxymorphone, but without changes to analgesia or toxicity [30].

### Pharmacogenomics for stronger opioids

Clinical utility refers to the capacity of a genetic test to improve clinical outcomes [57]. Although several actionable gene variants have been identified for codeine and tramadol drug-gene pairs, their clinical utility is limited, as these are weak opioids less commonly initiated in the palliative care population. It is important to consider available evidence on the usual “stronger” opioids that this population requires.

Even though the choice of initial opioid prescription is made largely based on a trial-and-error approach, morphine is commonly considered a first choice opioid for cancer pain [58], in part due to its widespread availability and low cost, with oxycodone being a suitable alternative [59]. For example, an Australian study examining opioid prescriptions under its government subsidy program found that cancer patients were 2.34 times more likely to have morphine initiated than oxycodone [60]. Furthermore, the availability of several strong opioids (e.g. morphine, oxycodone, fentanyl, hydrocodone, hydromorphone, and methadone) has made opioid switching common practice, where if one opioid results in inadequate analgesia or intolerable adverse effects, it is ceased and a different opioid is trialled [61]. Population-based studies have generally failed to detect superiority of any one opioid for cancer pain [62–65]. However, opioid rotation is common (20–44%), and when it is required, it often improves analgesia and reduces adverse effects, highlighting that for a certain cohort of patients, there may be an individualised “superior” opioid, the genetic basis of which is unknown [66–70].

Hydrocodone is the commonest opioid used in the USA, has actionable pharmacogenomic variants [30] but is unavailable in most countries [71]. CYP2D6 IMs and PMs have reduced capacity to metabolise hydrocodone into hydromorphone, its more active metabolite. Although the pharmacokinetic dose-response is clearer, it is unclear whether this translates to clinical differences in analgesia or toxicity. CPIC therefore recommends a usual conservative approach of using hydrocodone, whereby if there is no analgesic effect, to consider an alternative opioid not metabolised by CYP2D6 (i.e. not codeine, tramadol, or oxycodone) [30]. Data on UMs are not yet sufficient to guide prescribing [30].

Methadone is a more widely available opioid that requires CYP2B6 for conversion into inactive metabolites [72]. There is a moderate level of association between methadone and CYP2B6, where *1 and *4 alleles increase methadone clearance, and *6 and *18 cause decreased methadone clearance [30]. However, this is mainly limited to the pharmacokinetic dose-response in patients prescribed methadone for harm minimisation in heroin addiction [73–76], and thus, there is no current prescribing analgesic recommendation [30]. This nonetheless remains a promising area for future research.

### Other important genes

Although currently available evidence for pharmacogenomic-guided opioid selection among stronger opioids is less robust, there are several noteworthy genes that should be discussed, pending future validation. Opioid receptor mu 1 (*OPRM1*) and catechol-O-methyltransferase (*COMT*) are pharmacogenes with weak evidence related to opioid prescription to date, even though they have been studied in various populations and in all the strong opioids [30].

*OPRM1* encodes the mu opioid receptor, the target site of all opioid analgesics [14]. It is responsible for analgesia and its side effects (constipation, sedation, respiratory depression) [14]. *OPRM1* has a functional SNP (rs1799971), which is most common in East Asians (e.g. Japanese, Chinese, and Koreans) (49%), compared with Europeans (15%) and African Americans (5%) [77]. This variant is located in the protein-coding part of the *OPRM1* gene and replaces the normal amino acid at residue 40, asparagine (Asn), with aspartic acid (Asp) [78]. Presently, although this variant is associated with increases in morphine requirements, its effect is usually marginal and does not translate into clinically significant dose alterations especially in studied European groups [30]. Most studies were in a postoperative setting, and there is no clarity as to the effects of the common confounders in pain response, or its significance in cancer pain. There are conflicting assertions regarding the effect of this variant on fentanyl dosing, and for other common opioids (buprenorphine, codeine, hydrocodone, hydromorphone, methadone, oxycodone, tramadol), there is either insufficient evidence or no effect seen at present [30].

Pain is also modulated through catecholamines (dopamine, adrenaline, and noradrenaline), which affect pain sensitivity and enhance opioid analgesic effects [2]. COMT encodes for an enzyme, which metabolises and inactivates catecholamines and breaks down dopamine in the brain [79]. COMT has a functional SNP (rs4680), which causes a valine to methionine amino acid substitution at residue 158 in the protein sequence (Val158Met). This SNP alters the structure of the enzyme by substituting the wild-type allele (G) (which codes for a valine amino acid) to (A) (which codes for methionine) [79]. This leads to a three- to fourfold reduction in COMT enzyme activity, leading to greater dopamine levels in the brain, and is associated with favourable analgesic response to opioid use, and variations in pain perception [80].

These studies require further validation and replication in clinical settings to strengthen their clinical validity. However, they represent promising biomarkers that may influence pain and opioid response. Other gene variants that may contribute to opioid response are currently being studied [25], but more work is needed to clinically validate any associations and to inform prescribing. Thus far, most genetic studies of this nature have been conducted in Caucasian (European ancestry) populations; future clinical studies are important in ethnically diverse groups to establish variant frequencies and the potential for clinical impact at the population level.

### How can pharmacogenomics be helpful to guide opioid prescription?

Opioid prescription is currently based on a combination of objective factors (e.g. renal and liver function, location and mechanism of pain), subjective factors (subjective pain experience, psychosocial elements), and past history (comorbidities, opioid exposure, addictive behaviour). Although opioid titration should always be driven by clinical response, pharmacogenomic parameters will provide the clinician with additional and upfront confidence in deciding which opioids to preference or avoid, and provide reassurance on the speed at which these opioids may be titrated to deliver optimal analgesia as quickly as possible. This could mitigate the need for switching between multiple potentially ineffective or intolerable opioids, thus reducing suffering, time, and healthcare costs.

Internationally and across clinical settings, physicians are already prescribing certain medications based on routine pharmacogenomic testing. An example is the testing of variants determining thiopurine methyltransferase (TPMT) activity to predict thiopurine containing medications in rheumatology [81], which balances risk (marrow toxicity) and frequency of TPMT deficiency (0.3% of population) [81].

## Current limitations to pharmacogenomic-guided prescribing

Although there is clinically actionable evidence relating to pharmacogenomics and opioids, questions remain around the cost-benefit of real-time or prospective CYP2D6 genotyping. Ideally, pharmacogenomic testing would occur prior to treatment to guide decision making. However, in reality, it is likely that pharmacogenomic testing may occur after referral to palliative care teams, meaning patients may have already suffered toxicity from potentially unsuitable opioids. In this scenario, PGx testing could still be useful as means to retrospectively explain adverse reactions or lack of efficacy, and provide more confidence in downstream treatment decisions. With the current state of evidence, the proportion of patients that will benefit is unclear, and this depends on whether the test is being performed for all actionable variants or only specific variants of interest.

Currently, opioid pharmacogenomic tests are usually either self-funded or accessed through research studies. Most clinicians have limited expertise in interpreting pharmacogenomic tests results, advising patients on testing locations, or understanding current pharmacogenomic data [16]. As opioid pharmacogenomic testing is not current standard of practice, self-funded tests may be difficult to organise, especially without a clear pathway of testing specific variants of interest using accredited laboratories. Depending on local practice, the appropriate pathway may involve an initial referral to a genetics service, or a pharmacology service, or even directly to an accredited laboratory. Finally, the turnaround time of several weeks needs to be taken into consideration with timing the test for optimal clinical benefit. There are opportunities for palliative care to mirror the steps taken in progressing the field of personalised oncology, namely to facilitate opioid pharmacogenomic testing through research programs and simultaneously educate and upskill clinicians in the field.

## Conclusion — now and in the future

Pharmacogenomic evidence underpinning and influencing opioids prescribing is currently limited to codeine and tramadol. However, research is accelerating in this area with other opioids, where oxycodone (CYP2D6) and methadone (CYP2B6) already have moderate evidence of pharmacogenetic associations with drug response, and with a number of emerging genes, some studied in more opioids than others. These are promising results which require clinical validation, and it is possible that international guidelines will soon expand in this area. The increasingly rapid turnover and reducing costs of pharmacogenetics tests means greater accessibility and affordability to patients. Clinicians will be increasingly asked questions about this and be asked to provide information and guidance about this area of care.

Based on the current evidence, if seeking to incorporate pharmacogenomic testing into opioid prescribing practice, such testing should focus on the CYP2D6 gene and its actionable variants. Pharmacogenomics is an important possible area of expansive knowledge, and palliative care, like other areas of medicine, will increasingly be influenced by and influence this body of science. The challenge for those working at the intersection of palliative care and pharmacogenomics is to ensure key findings that influence practice are communicated in a manner that are readily accessible to clinicians as they navigate this emerging area of care.
